# Editorial: Neurodegeneration, cell signaling and neuroreparative strategies, Volume II

**DOI:** 10.3389/fphar.2023.1198151

**Published:** 2023-04-12

**Authors:** Ana Rita Vaz, Ana Sofia Falcão, Valle Palomo

**Affiliations:** ^1^ Neuroinflammation, Signaling and Neuroregeneration Group, Research Institute for Medicines (iMed.ULisboa), Faculty of Pharmacy, Universidade de Lisboa, Lisbon, Portugal; ^2^ Department of Pharmaceutical Sciences and Medicines, Faculty of Pharmacy, Universidade de Lisboa, Lisbon, Portugal; ^3^ Faculdade de Ciências Médicas, iNOVA4Healt, NOVA Medical School, NMS, FCM, Universidade Nova de Lisboa, Lisboa, Portugal; ^4^ Instituto Madrileño de Estudios Avanzados en Nanociencia (IMDEA-Nanociencia), Madrid, Spain; ^5^ Centro de Investigación Biomédica en Red de Enfermedades Neurodegenerativas (CIBERNED), Instituto de Salud Carlos III, Madrid, Spain

**Keywords:** neurodegenerative diseases, CNS-intracellular and extracellular communication, neuroinflammation and immunoregulation, therapeutic applications, hypoxic-ischemic injury, neurotoxicity

Neurodegeneration is characterized by distinct molecular mechanisms, including protein misfolding, endoplasmic reticulum stress, mitochondria and proteasome deregulation, and immunoderegulation. These malfunctions accounts for the progressive death of neuronal and non-neuronal cells in specific regions of the central nervous system (CNS). These processes are not only found in classical neurodegenerative diseases, but also in familial or sporadic neurodevelopmental disorders, as well as in other CNS-associated damage that can be caused by environmental threats. A better understanding of the role of each cell type and their contribution for such neurodegeneration, with the proper identification of the cellular and molecular pathways involved, will contribute to better clarify the pathophysiological mechanisms that trigger neurodegeneration in order to find more precise targets for modulation.

This Research Topic gives an overview of the neuronal-glial pathophysiological mechanisms involved in several existing models of brain damage, including neuroinflammation in inflammatory pain, hypoxic-ischemic injury, Alzheimer’s Disease (AD) and toxicity caused by methylmercury, through 6 articles by 44 authors, which contains 1 mini-review and 5 original research papers (total views: 4,334; as of 24 March 2023). It also gives new insights on the pharmacological and clinical relevance of the research involving cannabinoid receptors. A particular attention is given to the discovery of a subset of genes for the early diagnosis and disease progression prediction of AD, as well as the new potential therapeutic strategies that are being developed for this disease.

One of the studies approaches the Cannabinoid receptor type 1 (CB1), a G-protein coupled receptor, that is the main CNS cannabinoid receptor and is implicated in several brain functions such as long-term potentiation (LTP) and other forms of activity-dependent plasticity, having a role in the metabolic regulation, pain and anxiety, among other CNS processes (Goldberger et al.). The Authors show the first experimental evidence that the voltage dependence is different for the two main endocannabinoids, 2-AG and anandamide, suggesting that this regulatory modality may have physiological implications. Interestingly, they showed that the phytocannabinoid Tetrahydrocannabinol (THC), the main psychoactive component of the Cannabis plant, activates the CB1 receptor in a voltage dependent manner, which adds on the understanding of physiological functions mediated by the endocannabinoid system upon exposure to this substance. Another study identifies the pathways involved in acute toxic effects caused by methylmercury in the BV-2-microglial cell line when these cells are under an inflammatory insult (Martins et al.). In this study, the Authors demonstrate that the exposure of microglia to high levels of methylmercury impairs their ability to promote a proper response when facing an inflammatory stimulus, namely in terms of production of reactive oxygen species and proinflammatory cytokines, while causing increased cell death by necrosis.

In addition, there are two original research studies devoted to potential therapeutic approaches for CNS-induced damage. One of them evaluates the benefits of the naturally extracted flavonol myricetin in the hypoxic-ischemic encephalopathy, using both *in vivo* and *in vitro* models, respectively, the modified Rice-Vannucci model (Vannucci and Vannucci, 2005) in male pups 7 days after birth and the PC12 cell line exposed to CoCl_2_ (Chen et al.). The Authors demonstrate that treatment with myricetin significantly reduces brain infarction size, glia activation, apoptosis, and oxidative stress through activation of the NRF2 (Nuclear factor-E2-related factor 2). It also increases the downstream antioxidant enzymes NQO-1 and HO-1. Overall, they point myricetin as a promising therapeutic agent for hypoxic-ischemic encephalopathy that acts *via* NRF2 signaling pathway. The other study evaluates the mechanisms underlying the benefits of the iridoid glycoside catalpol for inflammatory pain. For that, they use the rat model injected with complete Freund’s adjuvant (CFA) to produce an inflammatory pain model, either treated or not with catalpol (Zhao et al.). The Authors found that catalpol effectively reduced CFA-induced mechanical allodynia and thermal hyperalgesia, while regulating the HDAC4/PPAR-γ-signaling pathway in CFA rat spinal cord neurons. In addition, catalpol was able to reduce the NF-κB/NLRP3 inflammatory axis in the spinal cord of CFA rats and peripheral pain by inhibiting tissue inflammation.

Finally, there are two studies devoted to Alzheimer’s Disease. The first one explores the role of endoplasmic reticulum stress-related genes in AD patients based on interpretable machine learning (Lai et al.). In this study, the Authors demonstrate a correlation between endoplasmic reticulum stress and infiltrated immune cells, proposing six ER stress-related genes that may predict AD progression (RNF5, UBAC2, DNAJC10, RNF103, DDX3X, and NGLY1), based on nine machine learning algorithms. This study may provide novel targets for individualized treatment in patients with AD, and novel biomarkers to predict disease progression and early diagnosis. The other study is a mini-review that summarizes the dysregulation of the E3 ubiquitin ligase anaphase promoting complex/cyclosome that binds Cdh1 (APC/C-Cdh1) in the pathogenesis of AD, with a particular emphasis in the neurotoxicity induced by its molecular targets (Lapresa et al.). Indeed, dysfunction of the APC/C-Cdh1 complex can trigger dendrite disruption, synapse loss and neurodegeneration, leading to memory and learning impairment, which are considered hallmarks in the neurodegenerative process in AD. The Authors strengthen the importance of understanding the role of APC/C-Cdh1-targeted substrates in AD for the development of new effective disease-modifying treatments for this neurodegenerative disorder.

Overall, this Research Topic discussed a few proofs of concept that both neuronal and non-neuronal cells contribute to the degenerative process in different models of neurodegeneration and how the identification of their molecular mechanisms can be used as biomarkers of CNS damage. In addition, some modulatory strategies are here proposed as novel therapeutic strategies to overcome the neurodegenerative process.
